# Spatial niches of the colorectal cancer microbiome: differences, interrelationships, and clinical implications of fecal, mucosal, and intratumoral microbiota

**DOI:** 10.3389/fcimb.2026.1904188

**Published:** 2026-09-04

**Authors:** Minghui Yu, Xin Shi, Dong Li, Yuelin Li, Mingyu Sun

**Affiliations:** 1 Shuguang Hospital, Shanghai University of Traditional Chinese Medicine, Shanghai, China; 2 Shanghai University of Traditional Chinese Medicine, Shanghai, China; 3 Key Laboratory of Liver and Kidney Diseases, Institute of Liver Diseases, Shuguang Hospital, Shanghai University of Traditional Chinese Medicine, Shanghai, China

**Keywords:** colorectal cancer, fecal microbiota, gut microbiome, intratumoral microbiota, mucosal microbiota, non-invasive screening, precision medicine, spatial niche

## Abstract

Colorectal cancer (CRC) is one of the most common malignancies worldwide and a leading cause of cancer-related mortality. The gut microbiome has attracted growing attention because of its involvement in CRC initiation and progression, early detection, prognostic evaluation, and therapeutic response. However, CRC microbiome studies continue to face inconsistent findings and limited reproducibility, partly because of differences in specimen type. Directly comparing microbial signals from fecal, mucosal, and intratumoral samples may therefore bias mechanistic interpretation and mislead clinical translation. From a spatial-niche perspective, this review summarizes the distinct characteristics, formation mechanisms, interrelationships, and clinical implications of fecal, mucosal, and intratumoral microbiota during CRC progression. We further propose a “seed bank–colonizers–specialized populations” model to conceptualize the continuous but non-equivalent hierarchy among these niches. By providing a clearer spatially stratified framework, this review aims to help move CRC microbiome research beyond descriptive associations toward precision-oriented applications supported by mechanistic interpretability, reproducible evidence, and clinical translatability.

## 1 Introduction

Colorectal cancer (CRC) remains one of the most burdensome malignancies worldwide. According to GLOBOCAN 2022 global cancer statistics, CRC ranks among the leading causes of cancer incidence and mortality worldwide and continues to impose a substantial burden on public health systems (Bray et al., 2024). The rising incidence of early-onset CRC and the increasingly evident shift toward younger age at diagnosis have become major global public health concerns (Siegel et al., 2019; Sinicrope, 2022). The gut microbiome has attracted considerable attention because of its involvement in CRC initiation, progression, and therapeutic response. Accumulating evidence indicates that the gut microbiota contributes to epithelial barrier homeostasis, local inflammatory responses, metabolic remodeling, and immune regulation, and may also influence tumor initiation, progression, metastasis, and therapeutic response; accordingly, the microbiome has become a key focus in CRC early detection, prognostic stratification, and strategies to enhance therapeutic responsiveness (Sepich-Poore et al., 2021; Wong and Yu, 2023; White and Sears, 2024).

However, CRC microbiome studies continue to be challenged by inconsistent findings, limited reproducibility, and poor cross-study comparability. A major contributor to these challenges is heterogeneity in specimen types across studies. Some studies rely on fecal specimens, others use intestinal mucosal biopsies, and still others directly analyze tumor tissues or intratumoral microbiota. The gut microbiota exhibits pronounced spatial organization, and microbial communities across anatomical sites differ in their physicochemical microenvironments, proximity to host tissues, and pathological information encoded (Donaldson et al., 2016; McCallum and Tropini, 2024). Therefore, ignoring differences in specimen source and spatial hierarchy and directly comparing microbiome data from distinct ecological niches may bias mechanistic interpretation and mislead clinical translation (Donaldson et al., 2016).

Fecal samples are currently the most widely used specimens in CRC microbiome research and are preferred in many microbial biomarker studies because they are non-invasive, easy to collect and store, and suitable for longitudinal monitoring (Baxter et al., 2016; Zwezerijnen-Jiwa et al., 2023). Nevertheless, fecal microbiota primarily represent mixed luminal microbial communities shed from the colorectal lumen and are readily influenced by multiple factors, including diet, medication use, intestinal transit time, sample collection and storage procedures, and population background. Consequently, their limited spatial resolution makes it difficult to accurately capture localized microbial perturbations directly associated with tumor lesions (Tropini et al., 2017; Mehra and Kumar, 2024; Tito et al., 2024). In contrast, mucosa-associated microbiota colonize the intestinal mucus layer and epithelial surface, forming a critical interface for host–microbe interactions. Alterations in their community structure can be detected as early as the precancerous adenoma stage (Dejea et al., 2014; Nakatsu et al., 2015). Intratumoral microbiota, in turn, reside within tumor tissues and constitute an important component of the tumor microenvironment (TME). Within this niche, they may directly mediate biological processes such as DNA damage, immunosuppression, tumor invasion and metastasis, and therapeutic resistance (Geller et al., 2017; Yu et al., 2017; Nejman et al., 2020).

**Figure 1:**
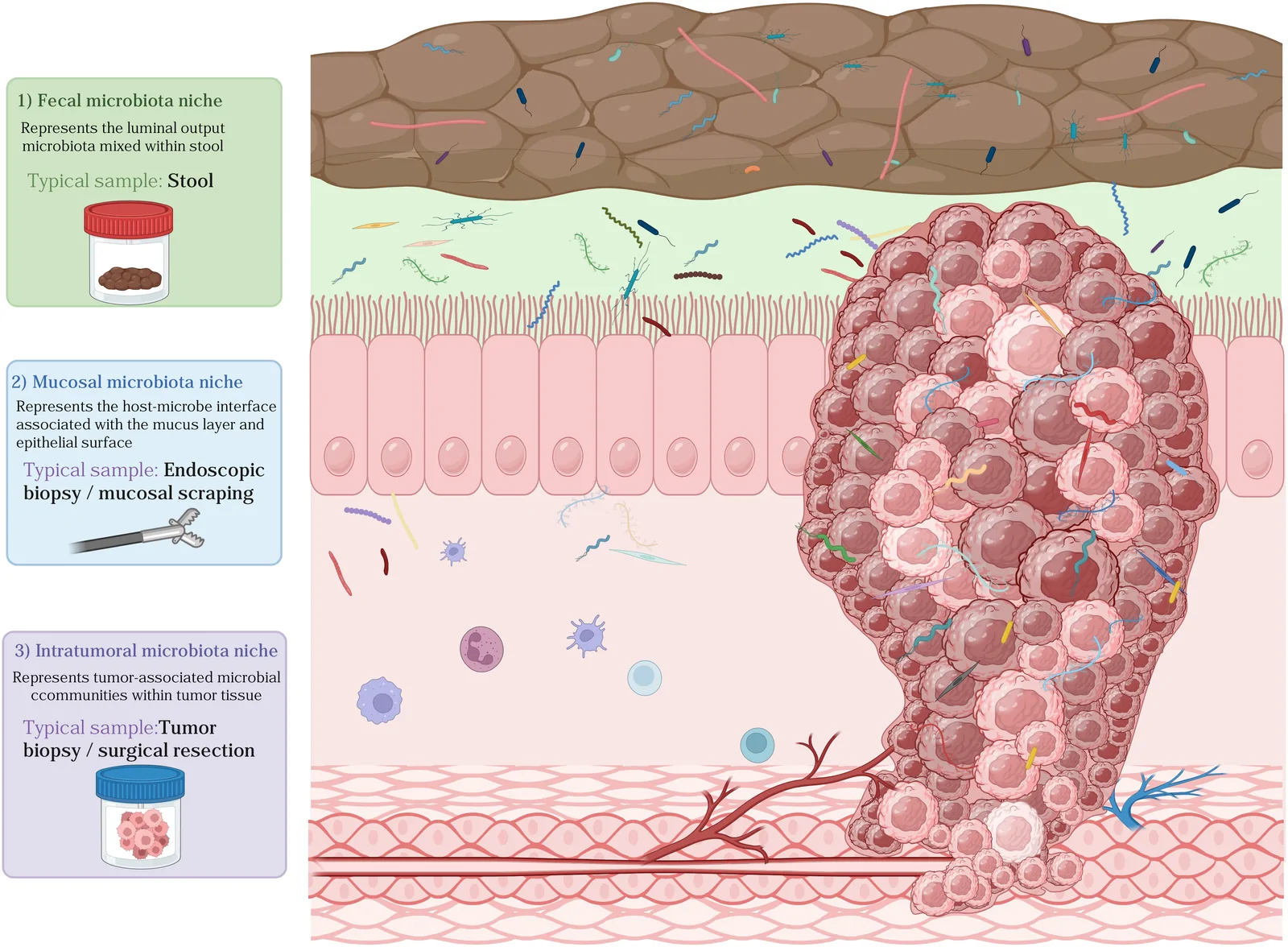
Spatial niches and representative specimen types in colorectal cancer microbiome research. The schematic illustrates three major microbiota-associated niches: the fecal microbiota niche, representing luminal microbial output; the mucosal microbiota niche, representing the host–microbe interface associated with the mucus layer and epithelial surface; and the intratumoral microbiota niche, representing tumor-associated microbial communities within the tumor microenvironment. Representative specimen types include stool, endoscopic biopsy or mucosal scraping, and tumor biopsy or surgical resection, respectively.

Therefore, fecal, mucosal, and intratumoral microbiota should not be regarded as simple reproductions of the same microecological signature across different specimen types. Rather, they represent distinct spatial information carriers corresponding to free luminal communities, the host–epithelial interaction interface, and microbial populations colonizing the TME, respectively. **Figure 1** illustrates these three spatial niches and their corresponding specimen types, highlighting the spatial progression from luminal output to the mucosal interface and ultimately to the intratumoral microenvironment. Ignoring the intrinsic differences among these ecological niches, integrating microbiome data across niches without spatial stratification, or simply extrapolating findings from fecal microbiota studies to mucosal or intratumoral contexts may bias interpretations of pathological mechanisms and reduce the translational value and reliability of downstream clinical applications. Against this background, this review uses spatial niches as an organizing framework to summarize the distinctive features, interrelationships, and potential clinical implications of fecal, mucosal, and intratumoral microbiota during CRC progression.

## 2 Characteristics and biological significance of the three spatial niches

### 2.1 Fecal microbiota: community signatures at the luminal output level

Fecal samples are among the most commonly used specimen types in CRC microbiome research because they are non-invasive, easy to collect, and suitable for repeated sampling. These features make them particularly applicable to large-scale population screening cohorts and longitudinal monitoring at the individual level (Zeller et al., 2014; Young et al., 2021; Khannous-Lleiffe et al., 2022). Fecal microbiota primarily reflect the microbial community characteristics of expelled luminal contents and, to some extent, provide an integrated readout of microbial output from the intestinal tract, particularly the distal colorectum (Genua et al., 2021; Du et al., 2022).

In patients with CRC, fecal dysbiosis is commonly observed and is characterized by the relative enrichment of *Fusobacterium nucleatum* (*F. nucleatum*), enterotoxigenic *Bacteroides fragilis* (*B. fragilis*), certain pathogenic *Escherichia coli* (*E. coli*) strains, and several oral-derived or inflammation-associated taxa, including *Streptococcus*, *Peptostreptococcus*, *Parvimonas*, and *Gemella* (Loftus et al., 2021). Conversely, beneficial short-chain fatty acid-producing commensals are markedly depleted, suggesting a reduction in protective metabolic capacity within the gut (Du et al., 2022; Zhang et al., 2022; Cai et al., 2025). Beyond taxonomic alterations, fecal samples from patients with CRC also exhibit perturbations in metabolic pathways related to amino acid metabolism, bile acid metabolism, short-chain fatty acid production, and inflammation, suggesting impaired ecological homeostasis at the luminal level (Le Gall et al., 2018; Kim et al., 2020).

Importantly, feces represent an integrated terminal readout of luminal contents across the intestinal tract, providing a broad overview of intestinal dysbiosis rather than fully reflecting the *in situ* microbial status on the mucosal surface or within tumor tissues. Studies using paired specimens have demonstrated compositional and functional differences between fecal microbiota and mucosal or tumor-associated microbiota. Some lesion-associated taxa show fecal signals that differ in magnitude from those observed at the disease site (Flemer et al., 2017; Wirth et al., 2020; Yin et al., 2024; Kim et al., 2025). Therefore, microbial alterations detected in feces should not be directly equated with the actual *in situ* colonization of these microorganisms on the mucosal surface or within tumor tissues. Moreover, confounding factors such as intestinal motility, diet, medication exposure, comorbidities, and age may substantially affect the compositional and functional profiles of fecal microbiota (Loftus et al., 2021; Adnan et al., 2024; Fusco et al., 2024).

Owing to their relatively low spatial resolution, fecal samples have limited ability to identify which microorganisms truly colonize the lesion surface or reside within tumor tissues. They also cannot independently determine whether these microorganisms directly participate in local carcinogenic mechanisms. Therefore, fecal microbiota are better suited as a practical entry point for non-invasive screening and longitudinal monitoring than as direct surrogates for mucosal or intratumoral microbiota (Krigul et al., 2021; Young et al., 2021; Khannous-Lleiffe et al., 2022; Brezina et al., 2023).

### 2.2 Mucosal microbiota: a host–microbe interface niche

Mucosa-associated microbiota primarily colonize the intestinal mucus layer and epithelial surface, forming a critical host–microbe interface that separates luminal contents from host tissues. Compared with fecal communities, this niche exhibits greater spatial selectivity in microbial colonization. The outer intestinal mucus layer is relatively loose, allowing certain bacteria to adhere and establish stable colonization, whereas the inner mucus layer is more densely organized and regulated by antimicrobial peptides, secretory IgA, and local immune-cell surveillance (Johansson et al., 2008, 2011; Vaishnava et al., 2011).

Accordingly, mucosal microbiota should not be regarded as proximal replicas of fecal microbiota, but rather as interface-specific communities shaped by adhesive capacity, immune selection, and adaptation to local nutrient conditions (Wirth et al., 2020; Yin et al., 2024; Kim et al., 2025). During CRC initiation and progression, intestinal mucosal barrier disruption, intensified local inflammation, and epithelial metabolic reprogramming may further promote the enrichment of specific pathobionts within this niche. Taxa such as *F. nucleatum*, *Campylobacter*, *Parvimonas*, *Peptostreptococcus*, and *Granulicatella* are frequently enriched in mucosal tissue samples from patients with CRC, and abnormal enrichment of some species may emerge as early as the precancerous adenoma stage (Nakatsu et al., 2015; Shaw et al., 2021; Zhang et al., 2021a; Zhao et al., 2021; Avelar-Barragan et al., 2022; Costa et al., 2022).

The unique value of mucosal microbiota lies in their ability to more directly reflect pathophysiological changes at the host–microbe interface. Pro-carcinogenic taxa, including *F. nucleatum*, *B. fragilis*, and pks-positive *E. coli*, may affect the epithelial barrier and local immune microenvironment through adhesive colonization, β-catenin signaling activation, Th17-associated inflammation, colibactin-mediated genotoxic injury, and mucosal biofilm formation, thereby contributing to colorectal adenoma-to-carcinoma progression (Wu et al., 2009; Arthur et al., 2012; Rubinstein et al., 2013; Dejea et al., 2018; Nardelli et al., 2021; Han et al., 2026). Moreover, mucosal samples are more likely than fecal samples to capture locally colonizing taxa closely associated with CRC initiation and progression. They may also help reveal key features such as altered host intestinal epithelial gene expression, disruption of the Th17/Treg immune balance, and abnormalities in the local metabolic microenvironment (Campillo-Gimenez et al., 2021; Chen et al., 2022; Vilkoite et al., 2026).

Therefore, mucosal samples are particularly suitable for addressing key questions related to colorectal precancerous lesion development, local inflammation, intestinal barrier dysfunction, and mechanisms of microbe–host interactions at the mucosal interface. However, their limitations should also be recognized. Sampling typically relies on endoscopic brushing, mucosal scraping, or tissue biopsy, which are invasive or minimally invasive procedures. In addition, substantial heterogeneity in mucosal microbiota across intestinal segments and lesion sites complicates sampling standardization and limits their implementation in large-scale population screening (Tajima et al., 2022).

### 2.3 Intratumoral microbiota: specialized communities within the tumor microenvironment

Intratumoral microbiota comprise low-biomass microbial populations or signals detected within tumor tissues and are commonly conceptualized as a highly specialized niche shaped by the selective pressures of the TME, including hypoxia, acidity, nutrient competition, immunosuppression, and metabolic heterogeneity. Compared with fecal or mucosa-associated communities, tumor tissue-associated microbial populations may reflect additional selection and retention within malignant tissues. Consequently, only a restricted subset of taxa may be capable of long-term persistence in this environment, and spatially validated communities may interact more directly with tumor and immune cells and potentially influence therapeutic responses (Long et al., 2024; Zong et al., 2024).

Evidence indicates that *F. nucleatum*, *B. fragilis*, pks-positive *E. coli*, and certain oral-derived anaerobes may be enriched in CRC tumor tissues or tumor-associated microenvironments (Toprak et al., 2006; Castellarin et al., 2012; Kostic et al., 2012; Boleij et al., 2015). However, emerging evidence suggests that intratumoral microbiota should not be interpreted solely on the basis of taxonomic enrichment. Instead, their biological significance depends on spatial localization, microbial activity, and interactions with host cells within the tumor ecosystem. Recent advances in spatial imaging, *in situ* detection, and multi-omics approaches have revealed spatially organized tumor-associated microbial patterns linked to tumor cell states, immune-cell infiltration, and local metabolic characteristics (Wang et al., 2024; Zhang et al., 2024; Liao et al., 2025). Whole-transcriptome profiling of 807 CRC tumors further showed that oral-derived taxa, including several *Fusobacterium* species, were enriched in right-sided, MSI-H, and BRAF-mutant tumors. Notably, associations between *F. animalis* and host collagen-related and immune pathways were context-dependent and were most prominent in CMS4 tumors (Younginger et al., 2023). A large-cohort tissue-profiling study using a stringent decontamination workflow further identified anatomically and molecularly stratified tissue-resident microbial signatures and showed that microbial risk scores derived separately from tumor tissues and normal adjacent tissues predicted patient prognosis independently of established clinico-molecular factors (Shi et al., 2025b). Integrated analyses of microbiome, immune, and molecular features in tumor tissues also showed that microbial signatures were associated with tumor immune phenotypes and clinical outcomes (Roelands et al., 2023).

These intratumoral or tumor-associated microbes may contribute to malignant progression through multiple mechanisms, including activation of tumor-cell proliferative signaling, induction of genotoxic damage, shaping of local inflammatory or immunosuppressive microenvironments, and modulation of drug metabolism and therapeutic response (Nougayrède et al., 2006; Arthur et al., 2012; Rubinstein et al., 2013; Dejea et al., 2018; Dai et al., 2025; Yu et al., 2025; Sorrenti et al., 2026). A recent metatranscriptomic analysis revealed functional remodeling of tumor tissue-associated microbial communities, including a shift from carbon metabolism toward amino-acid-related functions, increased lipopolysaccharide biosynthesis and transport, and enhanced expression of *F. nucleatum* virulence factors. The co-activity of *F. nucleatum* with *Parvimonas micra*, *Peptostreptococcus stomatis*, *Granulicatella adiacens*, and *Hungatella hathewayi* further suggests that tumor-associated microbial functions may arise from cooperative networks rather than single taxa acting independently (Buetas et al., 2026).

Compared with fecal and mucosal microbiota, intratumoral microbiota are more closely aligned with the core functional microenvironment of CRC lesions. In this review, the term “intratumoral microbiota” is used operationally to refer to microbial populations or signals detected in tumor specimens. Within this broad category, we distinguish spatially validated intratumoral microorganisms, supported by *in situ* localization, imaging, or other orthogonal evidence, from broader tumor-associated microbiota or tissue-resident microbial signatures inferred from appropriately controlled bulk DNA or RNA sequencing. The latter should be interpreted as putative tumor tissue-associated microbial communities rather than direct evidence of active intratumoral colonization or functional involvement. These populations may reflect potential ecological adaptation and functional enrichment under the selective pressures of the TME (Dohlman et al., 2021; Galeano Niño et al., 2022; Long et al., 2024; Zhang et al., 2024). Nevertheless, this field remains highly controversial. Intratumoral microbial biomass is extremely low, interference from host genomic material is substantial, and samples are highly vulnerable to exogenous contamination from laboratory reagents and sample processing procedures. Even when microbial signals are detected by sequencing, it remains essential to determine whether they represent active colonizers, passive passengers, or technical noise (Wang et al., 2024; Zong et al., 2024; Li et al., 2026).

Therefore, conclusions regarding intratumoral microbiota should not rely solely on bulk sequencing data but should instead be interpreted in conjunction with rigorous negative controls, decontamination workflows, *in situ* validation, spatial localization, and, where appropriate, functional characterization. Despite these technical bottlenecks, the intratumoral niche remains one of the most promising dimensions for mechanistic investigation and clinical translation, particularly for identifying which microorganisms truly participate in tumor progression, therapeutic response, and the emergence of treatment resistance (Murota and Jobin, 2021; Xie et al., 2025).

### 2.4 Comparative overview of the three specimen types

Overall, fecal, mucosal, and intratumoral microbiota constitute a progressively layered spatial continuum, extending from luminal output to the host–microbe interface and ultimately to the tumor core. These three specimen types differ substantially in sampling methods, microenvironmental features, ecological representativeness, advantages, limitations, and clinical applicability. Fecal sampling is non-invasive and repeatable, although its ability to localize lesion-associated microbial signals is limited. Mucosal sampling more closely reflects lesion-adjacent microenvironments; however, it relies on invasive or minimally invasive procedures. Intratumoral sampling is closest to the lesion core, yet its application is strongly constrained by low microbial biomass and the need for stringent contamination control (Lauricella et al., 2025). These niches also differ in microenvironmental pressures, proximity to host-cell interactions, community representativeness, and clinically relevant use cases. Therefore, when interpreting CRC-associated microbial signals, the key question is not which specimen type is “better,” but which scientific question each specimen type is best suited to address. To facilitate comparison, **Table 1** summarizes the major characteristics of fecal, mucosal, and intratumoral microbiota as three spatial niches.

**Table 1:** Systematic comparison of fecal, mucosal, and intratumoral microbiota as three spatial niches.

Specimen type	Spatial niche	Main sampling approach	Main information reflected	Key strengths	Main limitations	Representative references
Feces	Luminal output layer	Stool collection; FIT/gFOBT-derived samples	Integrated luminal dysbiosis; non-invasive biomarker signals	Non-invasive; repeatable; suitable for screening and longitudinal monitoring	Low spatial resolution; affected by diet, medication, transit time, and sample handling	(Zeller et al., 2014; Baxter et al., 2016; Flemer et al., 2017; Tropini et al., 2017; Young et al., 2021; Khannous-Lleiffe et al., 2022; Zwezerijnen-Jiwa et al., 2023)
Mucosa	Host–microbe interface	Colonoscopic biopsy, brushing, scraping, or mucus-associated sampling	Mucosa-adherent communities; barrier status; immune selection; potential biofilm formation	Closer to local lesions; suitable for mechanistic studies of early carcinogenesis and field effects	Invasive or minimally invasive; affected by sampling site, mucus adherence, and mucosal heterogeneity	(Johansson et al., 2008, 2011; Nakatsu et al., 2015; DeDecker et al., 2021; Avelar-Barragan et al., 2022; Tajima et al., 2022)
Intratumoral tissue	Tumor microenvironment	Surgical or biopsy-derived tumor tissue; spatial/*in situ* validation when feasible	Putative tumor-associated microbial communities; TME-related immune, metabolic, and therapeutic-response signals	Closest to tumor cells and immune microenvironment; useful for prognosis and treatment-response studies	Low microbial biomass; high contamination risk; requires negative controls and decontamination workflows	(Nejman et al., 2020; Dohlman et al., 2021; Galeano Niño et al., 2022; Hilmi et al., 2023; Long et al., 2024; Zong et al., 2024; Li et al., 2026)

CRC, colorectal cancer; FIT, fecal immunochemical test; gFOBT, guaiac fecal occult blood test; TME, tumor microenvironment.

These specimen types provide complementary rather than equivalent spatial information.

These specimen types do not form a simple linear hierarchy of superiority. Rather, they provide complementary observational windows for different research questions. Fecal samples are well suited for population screening, risk assessment, and longitudinal monitoring. Mucosal samples are closer to local lesions and are suitable for investigating early carcinogenesis, mucosal barrier disruption, biofilm formation, and cancer-adjacent field effects. Intratumoral samples are closest to the lesion core and provide more direct biological evidence for dissecting pro-tumorigenic mechanisms, prognostic stratification, and therapeutic response.

Accordingly, the roles of these three specimen types in CRC microbiome research should be clearly defined. When the research goal is non-invasive screening, risk prediction, or postoperative monitoring, fecal samples have greater translational potential. When the focus is the biological characterization of local lesions, mucosal samples offer greater specificity. When the aim is to elucidate relationships between the tumor microenvironment and functional endpoints, intratumoral samples can provide critical information.

A mechanistically informative and translationally meaningful study design should not seek a single “optimal” specimen type. Instead, it should establish a stratified sampling strategy aligned with the research objective and, whenever feasible, incorporate paired fecal, mucosal, and intratumoral sampling within the same individual to distinguish shared cross-niche signals from site-specific signals. Research conducted within such a spatially stratified framework can reduce the risk of misinterpreting broad fecal associations as local pathogenic mechanisms. It can also help avoid directly extrapolating mechanistic signals identified in mucosal or intratumoral niches into non-invasive biomarkers for population screening.

## 3 Formation of spatial stratification: from descriptive differences to mechanistic interpretation

### 3.1 Mechanisms underlying luminal–mucosal differentiation: selection, adhesion, immunity, and metabolic adaptation

Differences between luminal and mucosal microbiota are not merely artifacts of sampling site; instead, they largely reflect niche-specific selection shaped by distinct microenvironmental pressures. Luminal contents are generally anaerobic, relatively rich in diet-derived substrates, and continuously influenced by intestinal motility and content flow; therefore, they primarily reflect the overall output of free-living or transient commensal communities (McCallum and Tropini, 2024). In contrast, the mucosal interface is shaped by selective pressures imposed by the mucus barrier, local oxygen gradients, host-derived glycoproteins, secretory IgA coating, antimicrobial peptide secretion, and innate immune surveillance (Johansson et al., 2008, 2011; Vaishnava et al., 2011; Rogier et al., 2014). Microbes with adhesive and colonization capacity, oxidative stress tolerance, mucin-utilization ability, or synergistic interactions with neighboring microbial communities are more likely to establish stable residence within the mucosal microenvironment (Lu et al., 2016; Saffarian et al., 2019; Duncan et al., 2021).

This microecological differentiation is reflected in both the spatial localization and functional characteristics of microbial communities. Luminal microbes rely more heavily on diet-derived substrates and cross-community metabolic cooperation to maintain ecological stability, whereas mucosa-colonizing communities must adapt to host-derived glycoproteins, micro-oxygen gradients, and local immune selection pressures (Coker et al., 2022). Accordingly, even identical or closely related microbial taxa may differ between fecal and mucosal niches in relative abundance, metabolic activity, and modes of host interaction (Yilmaz et al., 2026).

During CRC initiation and progression, mucosal ecological selection may further promote the organization of microbial communities into biofilm structures (Johnson et al., 2015; Tomkovich et al., 2019). Invasive polymicrobial biofilms may cover the tumor surface and extend into adjacent non-tumorous mucosa, suggesting that mucosal microecological abnormalities are broadly associated with tumor lesions rather than representing isolated events (Dejea et al., 2014; Queen et al., 2025). Representative tissue-based data provide quantitative support for the involvement of biofilm organization in CRC. Invasive polymicrobial biofilms were detected in 89% of right-sided lesions, including 13 of 15 CRCs and all 4 adenomas examined, but in only 12% of left-sided lesions, including 2 of 15 CRCs and none of the 2 adenomas. Notably, all patients with biofilm-positive tumors also harbored biofilms on distant tumor-free mucosa. Biofilm-positive mucosa showed reduced epithelial E-cadherin, enhanced IL-6/STAT3 activation, and increased crypt epithelial proliferation (Dejea et al., 2014). In familial adenomatous polyposis, mucosal biofilms were enriched in colibactin-producing *E. coli* and enterotoxigenic *B. fragilis*. In tumor-prone mice, co-colonization with these strains increased colonic IL-17 responses and epithelial DNA damage, accelerated tumor onset, and increased mortality compared with colonization by either strain alone (Dejea et al., 2018). Recent fluorescence *in situ* hybridization and dual-RNA-seq analyses showed that biofilm-associated bacterial biomass and activity were associated with pro-inflammatory signaling and immune-cell infiltration in CRC tissues. Community-level bacterial activity showed stronger associations with the TME than individual core pathogens, underscoring the importance of spatial organization and collective microbial activity (Kvich et al., 2024). *F. nucleatum* and other oral-derived taxa appear to be preferentially enriched on mucosal and tumor surfaces (Castellarin et al., 2012; Komiya et al., 2019; Zwinsová et al., 2021; Ohashi et al., 2022). This phenomenon provides indirect support for an ecological continuum linking luminal exposure, mucosal selection, and stable interface colonization, although the precise temporal sequence requires further validation in longitudinal paired studies (Li et al., 2022).

### 3.2 Mechanisms underlying mucosal–intratumoral differentiation: barrier disruption, tissue invasion, and adaptation to the tumor microenvironment

Microecological differentiation from the mucosa to the intratumoral compartment involves a more stringent selection process than luminal–mucosal differentiation and depends more heavily on barrier disruption and increased tissue accessibility under pathological conditions. Colorectal adenomas and carcinomas are often accompanied by abnormal expression of barrier-related proteins and intensified local inflammation. This pathological context may enable microbial metabolites, and in some cases bacteria themselves, to cross the mucosal barrier and enter the tissue microenvironment (Grivennikov et al., 2012). Within this pathological context, certain microbial taxa may also exhibit more active adhesion and enrichment. For example, *F. nucleatum* can mediate adhesive colonization and specific recognition through its FadA and Fap2 proteins (Rubinstein et al., 2013; Gur et al., 2015; Abed et al., 2016). Extracellular vesicles derived from *F. nucleatum* have also been reported to be enriched in CRC and to promote bacterial adhesion, providing additional evidence for active colonization mechanisms (Zheng et al., 2024). Collectively, these findings suggest that the enrichment of intratumoral microbiota is not merely a passive deposition process but may involve actively regulated molecular mechanisms.

It should be emphasized, however, that microbial invasion and retention from the mucosa to the intratumoral compartment are unlikely to occur through a single route. Beyond the active adhesion and invasion mechanisms described above, they likely result from the combined effects of intestinal barrier disruption, receptor-mediated colonization, local inflammatory and immune pressures, and adaptive selection within the TME (Zhang et al., 2024; Liao et al., 2025). Once microorganisms enter tumor tissues, they must further tolerate harsh environmental pressures, including hypoxia, acidification, nutrient heterogeneity, and immunosuppression (Benej et al., 2024). Ultimately, only a small subset of microorganisms capable of adapting to these conditions can persist and establish stable colonization. These microorganisms may influence tumor progression through the release of virulence factors or metabolites, or through modulation of host signaling pathways (Chung et al., 2018; Zepeda-Rivera et al., 2024; Galeano Niño et al., 2026).

The biological effects of intratumoral microbial colonization involve both direct and indirect mechanisms. Direct effects include the regulation of tumor-cell proliferative signaling, cell-cycle progression, DNA damage responses, and genomic stability (Yang et al., 2017). Indirect effects are mainly mediated by remodeling of the local immune microenvironment and alterations in host metabolic networks (Liu et al., 2024; Chen et al., 2025b). These effects may include skewing myeloid-derived suppressor cells, neutrophils, and macrophages toward pro-tumor phenotypes (Sorrenti et al., 2026). In addition, microbiota-derived metabolites may influence T-cell function and sensitivity to clinical therapies (Xu et al., 2024).

Therapeutic interventions may further reshape these microecological selection pressures. Clinical interventions, including chemotherapy, radiotherapy, surgery, fecal stream diversion, and preoperative bowel preparation, may affect intestinal oxygen tension, mucus barrier architecture, microbial load, and local immune homeostasis, thereby reshaping microecological relationships among the intestinal lumen, mucosa, and tumor tissue (Lee et al., 2023; Benej et al., 2024). Some studies suggest that microbial taxa such as *F. nucleatum* can persist in primary and metastatic CRC lesions and influence responses to chemotherapy or radiotherapy (Bullman et al., 2017; Yu et al., 2017). A more cautious interpretation is therefore that therapeutic interventions may influence treatment sensitivity and ultimately increase the risk of resistance by reshaping the tumor-associated microecology. This process is likely bidirectional rather than a unidirectional, deterministic causal chain. Moreover, intratumoral microbiota may arise from multiple sources, including local microbial expansion, ectopic dissemination of oral-associated taxa, bacterial translocation following altered barrier or vascular permeability, co-retention with metastatic lesions, and secondary colonization at metastatic sites (Bertocchi et al., 2021; Ayabe and White, 2024). Thus, intratumoral microbiota are best understood as the dynamic outcome of multi-route entry, selective retention within the microenvironment, and subsequent functional adaptation.

### 3.3 An integrative model linking the three niches and defining the boundaries of current evidence

Taken together, current evidence suggests that the spatial stratification of CRC-associated microbiota can be conceptualized using a “seed bank–colonizers–specialized populations” model. Fecal microbiota primarily constitute an intestinal microbial reservoir, reflecting the integrated output of luminal microbial communities (Feng et al., 2015; Wirbel et al., 2019; Yachida et al., 2019). Mucosal microbiota represent interface-colonizing communities that can stably adhere and persist after multiple layers of host-interface filtering (Zhao et al., 2021). Intratumoral microbiota are highly specialized communities that are further selected and retained through the combined effects of intestinal barrier disruption, receptor-specific recognition, and selection pressures within the TME (Galeano Niño et al., 2022). This model helps explain several seemingly contradictory findings: some taxa may be detectable in feces yet may not directly participate in local pathogenic mechanisms at the lesion site. Conversely, other taxa may be markedly enriched in tumor tissues yet fail to generate fecal diagnostic signals of comparable magnitude or stability (Zhao et al., 2021; Liu et al., 2025).

Notably, some paired-sample studies suggest that the microbial profiles of cancer-adjacent mucosa or peritumoral tissues may be linked to both tumor tissues and distal normal mucosa, indicating that CRC-associated microecological abnormalities are not always strictly confined to the tumor lesion itself but may extend across adjacent mucosal regions as a microecological “field effect” (Liu et al., 2024). This further suggests that fecal, mucosal, and intratumoral niches are not isolated static sites; rather, they may form a continuous ecological axis extending from the intestinal lumen to the mucosa and then to the tumor core through stepwise selection.

**Figure 2:**
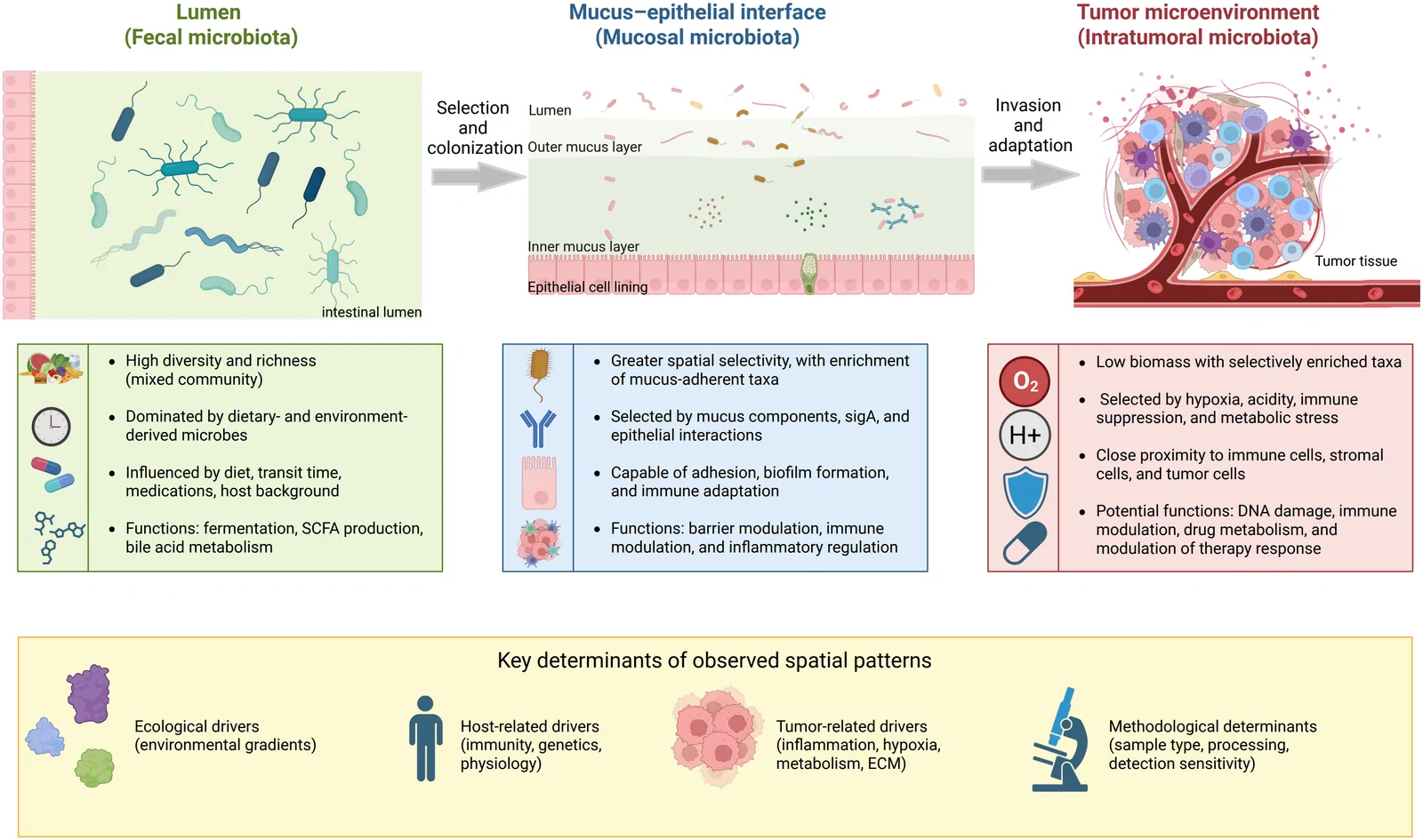
Mechanisms driving spatial stratification of the colorectal cancer microbiome. The schematic illustrates the ecological differentiation of CRC-associated microbiota across the luminal, mucus–epithelial, and tumor microenvironment niches, together with the major ecological, host-related, tumor-related, and methodological determinants of the observed spatial patterns. The arrows indicate conceptual transitions involving selection, colonization, invasion, and adaptation, rather than a universally established linear migration pathway. CRC, colorectal cancer; ECM, extracellular matrix; SCFA, short-chain fatty acid; sIgA, secretory immunoglobulin A.

However, this spatial stratification model should be viewed as a plausible theoretical framework rather than a fully validated linear migration pathway. Most existing studies still rely on cross-sectional analyses, case-control designs, or small paired cohorts, whereas studies that perform strain-level longitudinal tracking within the same patient and reconstruct temporal microbial dynamics remain scarce. Moreover, stronger evidence is currently concentrated on *F. nucleatum* and a limited number of pro-inflammatory or oral-derived taxa and remains insufficient for extrapolation to all CRC-associated microbiota (Nakatsu et al., 2025). Therefore, a more cautious interpretation at this stage is that current evidence supports a continuous yet non-equivalent hierarchical relationship among the three spatial niches, whereas the precise temporal patterns, generalizability, and interpatient heterogeneity of microbial cross-site migration still require validation through large-scale, multi-site paired analyses and longitudinal cohort studies (Baas et al., 2024; Xiang et al., 2026). To synthesize these mechanisms, **Table 2** summarizes the major ecological and pathological processes that may drive spatial stratification of the CRC microbiome across luminal, mucosal, and intratumoral niches. **Figure 2** complements this tabular summary by illustrating the progressive ecological differentiation across these niches, the principal ecological, host-related, and tumor-related drivers, and the methodological factors that may influence the observed spatial patterns. Accordingly, the arrows in **Figure 2** denote conceptual ecological transitions rather than proven directional migration between niches.

**Table 2:** Major mechanisms underlying spatial stratification of the CRC microbiome.

Mechanistic dimension	Main niche/transition	Core processes	Ecological outcome	Research implication	Representative references
Physicochemical gradients	Lumen–mucosa–intratumoral continuum	Oxygen, pH, nutrients, metabolites, and content flow	Niche-specific microbial selection	Explains cross-site microbial heterogeneity	(Donaldson et al., 2016; Coker et al., 2022; Benej et al., 2024; McCallum and Tropini, 2024)
Mucus barrier and adhesion	Lumen–mucosa transition	Mucin structure, mucin use, epithelial adhesion, and stable colonization	Enrichment of mucosa-adherent taxa	Distinguishes resident colonizers from transient luminal taxa	(Johansson et al., 2008, 2011; Lu et al., 2016; Saffarian et al., 2019; Duncan et al., 2021)
Host immune pressure	Mucosal and intratumoral niches	secretory IgA, antimicrobial peptides, inflammation, and immune-cell remodeling	Immune-selected microbial communities	Links dysbiosis to inflammation and immunosuppression	(Vaishnava et al., 2011; Grivennikov et al., 2012; Campillo-Gimenez et al., 2021; Galeano Niño et al., 2026; Sorrenti et al., 2026)
Biofilm formation	Mucosal and tumor surfaces	Polymicrobial aggregation, matrix formation, mucosal invasion, and surface persistence	Spatially organized local communities	Links biofilms to early lesions, tumor surfaces, and field effects	(Dejea et al., 2014; Johnson et al., 2015; Dejea et al., 2018; Tomkovich et al., 2019)
Tumor microenvironment selection	Intratumoral niche	Hypoxia, acidification, nutrient heterogeneity, immunosuppression, and metabolic stress	Putative tumor-associated microbial communities	Relates intratumoral microbiota to TME biology and therapy response	(Nejman et al., 2020; Galeano Niño et al., 2022; Benej et al., 2024; Long et al., 2024; Zong et al., 2024)
Cross-niche dissemination and retention	Lumen–mucosa–intratumoral continuum	Barrier disruption, tissue invasion, vascular leakage, dissemination, and selective retention	Shared but non-equivalent signals across niches	Supports paired sampling and cautions against direct extrapolation	(Grivennikov et al., 2012; Rubinstein et al., 2013; Bertocchi et al., 2021; Ayabe and White, 2024; Baas et al., 2024)

Spatial stratification should not be interpreted as a simple linear migration process, but rather as the result of ecological selection, host pressure, tumor-specific adaptation, and methodological constraints.

## 4 Clinical implications from the perspective of spatial niches

### 4.1 Clinical questions best addressed by different specimen types

From a clinical application perspective, fecal, mucosal, and intratumoral samples are not interchangeable diagnostic materials. Rather, they correspond to distinct layers of information in CRC microbiome research. The choice of specimen type fundamentally determines the clinical questions that a study can address. For example, fecal samples primarily reflect overall microecological output at the luminal level and are well suited for large-scale non-invasive screening, risk assessment, and longitudinal follow-up. Mucosal samples are closer to the host–microbe interface and are more appropriate for dissecting precancerous lesion progression, local inflammation, barrier dysfunction, and microecological differences among distinct carcinogenic pathways. Intratumoral samples, derived directly from the *in situ* TME, are particularly useful for exploring relationships between microbiota and tumor biology, immune status, prognostic stratification, therapeutic response, and resistance mechanisms (DeDecker et al., 2021; Dohlman et al., 2021; Avelar-Barragan et al., 2022; Hilmi et al., 2023).

Specifically, the major advantages of fecal samples include their non-invasive nature, ease of access, and suitability for repeated collection, making them a practical entry point for population screening and long-term follow-up monitoring. Their major limitation, however, is insufficient spatial resolution, which makes it difficult to determine whether the detected microbial signals truly originate from local lesions or tumor tissues (Zhou et al., 2022; Zouiouich et al., 2022). Although mucosal sample collection requires endoscopic procedures, these samples more directly reflect the microecological status of lesion-adjacent regions. They are therefore better suited for local validation of fecal screening signals and for mechanistic studies of ecological imbalance at the host–microbe interface during adenoma-to-carcinoma progression (DeDecker et al., 2021; Avelar-Barragan et al., 2022). Intratumoral samples are the most challenging to obtain and analyze, and low-biomass contamination must be rigorously controlled. Nevertheless, because they are spatially closest to tumor cells, immune cells, and the therapeutic microenvironment, they have unique value for prognostic assessment, prediction of therapeutic response, and elucidation of resistance mechanisms (Dohlman et al., 2021; Hilmi et al., 2023).

**Figure 3:**
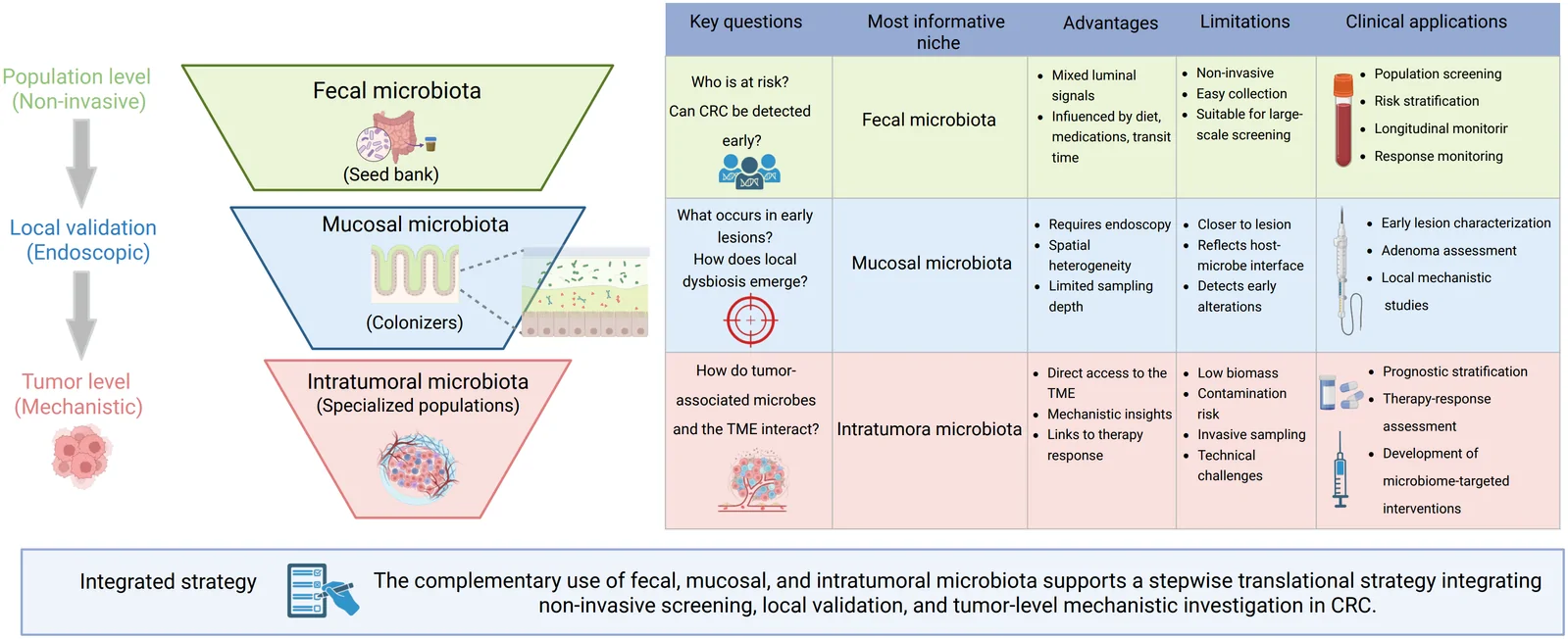
Clinical translation framework based on spatial niches of the colorectal cancer microbiome. The framework compares the research questions, advantages, limitations, and potential applications of fecal, mucosal, and intratumoral microbiota, and illustrates their complementary roles in non-invasive screening, local validation, and tumor-level mechanistic investigation. The inverted-pyramid arrangement represents increasing spatial specificity and mechanistic depth rather than the superiority of one specimen type over another. CRC, colorectal cancer; TME, tumor microenvironment.

Therefore, a more rational research and translational strategy is not to search for a single “best” specimen type, but to establish a stratified sampling framework in which non-invasive screening serves as the entry point, local validation serves as the bridge, and mechanistic interrogation serves as the core. **Figure 3** translates this principle into a spatial niche-based clinical framework by linking each specimen type to the research questions it is best suited to address, together with its advantages, limitations, and potential applications. In this framework, fecal microbiota support population-level screening, mucosal microbiota provide local endoscopic validation, and intratumoral microbiota support tumor-level mechanistic investigation and assessment of therapeutic response. These niches should therefore be viewed as complementary elements of a stepwise translational strategy rather than as competing specimen types. Integrated multi-niche analyses can leverage the distinct strengths of these specimen types to construct a coherent and mutually corroborating chain of microbiological evidence while reducing the limitations inherent to any single sampling approach. Following this stratified approach, **Table 3** summarizes the recommended specimen choices for major clinical and research scenarios in CRC microbiome studies.

**Table 3:** Clinical application scenarios and specimen selection in CRC microbiome research from a spatial niche perspective.

Clinical/research scenario	Preferred specimen type	Complementary data	Primary purpose	Key considerations	Representative references
Population screening	Feces	FIT/gFOBT; MT-sDNA; microbial/metabolite markers	Non-invasive screening	External validation; avoid causal inference from fecal signals	(Zeller et al., 2014; Baxter et al., 2016; Young et al., 2021; Khannous-Lleiffe et al., 2022; Brezina et al., 2023; Fan et al., 2023)
Risk stratification	Feces; mucosa in high-risk cohorts	Clinical factors; age; BMI; diet; medication; transit time; inflammation	Risk assessment and subgrouping	Control confounders and processing bias	(Alharfi et al., 2023; Adnan et al., 2024; Fusco et al., 2024; Mehra and Kumar, 2024; Tito et al., 2024)
Mechanisms of early carcinogenesis	Mucosa; adenoma or adjacent mucosa	Paired feces; epithelial/immune markers	Barrier dysfunction, biofilms, inflammation, and field effects	Consider site, lesion type, and pathway	(Dejea et al., 2014; Nakatsu et al., 2015; Dejea et al., 2018; DeDecker et al., 2021; Avelar-Barragan et al., 2022)
Tumor microenvironment studies	Intratumoral tissue	Spatial/*in situ* methods; immune/metabolic profiling	Microbe–tumor–immune interactions	Control low-biomass contamination; validate spatially	(Nejman et al., 2020; Dohlman et al., 2021; Galeano Niño et al., 2022; Hilmi et al., 2023; Wang et al., 2024)
Prognostic prediction	Intratumoral tissue; feces as supplement	Pathology; immune status; survival/recurrence data	Prognostic stratification	Requires independent validation	(Yu et al., 2017; Hilmi et al., 2023; Yu et al., 2025; Li et al., 2026; Sorrenti et al., 2026)
Prediction of treatment response	Intratumoral tissue or mucosa; serial fecal samples	Pre-/post-treatment samples; therapy outcomes	Resistance or sensitivity prediction	Association does not prove causality	(Bullman et al., 2017; Yu et al., 2017; Benej et al., 2024; Dai et al., 2025; Liao et al., 2025)
Microbiome-based intervention design	Feces, mucosa, or intratumoral tissue according to target niche	Multi-niche microbial, metabolic, immune, and response data	Target selection and niche-specific intervention	Luminal modulation may not affect local niches	(Bullman et al., 2017; Wang et al., 2023; Fan et al., 2025; Han et al., 2025; Liao et al., 2025; Tan et al., 2025)

BMI, body mass index; CRC, colorectal cancer; FIT, fecal immunochemical test; gFOBT, guaiac fecal occult blood test; MT-sDNA, multi-target stool DNA.

Sample selection should be driven by the clinical or mechanistic question rather than by convenience alone.

### 4.2 Implications for non-invasive screening and biomarker development

Early non-invasive screening for CRC is essential for reducing CRC-related mortality. From a spatial-niche perspective, a key principle for microbial biomarker development is that microbial signals detected in feces should not be simply equated with lesion-specific tumor signals. False-positive or false-negative results in screening based solely on fecal microbiota may arise, at least in part, not because the biomarkers are intrinsically ineffective, but because some microbial alterations genuinely associated with tumor initiation and progression are not adequately represented or effectively detected in fecal luminal output (Nejati et al., 2022; Alharfi et al., 2023; Brezina et al., 2023; Russo et al., 2023; Nikolić et al., 2025).

Therefore, future non-invasive screening should move beyond the identification of individual differentially abundant taxa and toward multi-target, multidimensional integration. On the one hand, fecal microbiota features can be integrated with host-derived fecal DNA methylation markers, metabolites, immune factors, and established tools such as the fecal immunochemical test (FIT) to construct host–microbe detection models with improved screening sensitivity and specificity (Baxter et al., 2016; Zhang et al., 2021b; Fan et al., 2023). Representative cohort data support the complementary value of microbiome-based screening rather than the replacement of FIT. In a cohort of 490 asymptomatic participants, a multitarget microbiota model combining stool bacterial abundances with fecal hemoglobin detected 91.7% of cancers and 45.5% of adenomas, compared with 75.0% and 15.7%, respectively, for FIT alone. Among lesions missed by FIT, the combined model identified 70.0% of cancers and 37.7% of adenomas. However, the increased sensitivity was accompanied by lower specificity than FIT alone (90.1% vs. 97.1%), highlighting the need for larger external validation studies before clinical implementation (Baxter et al., 2016). On the other hand, the correspondence between fecal biomarkers and local samples, including mucosal and intratumoral specimens, should be carefully examined to determine which fecal changes genuinely reflect abnormalities in mucosal or intratumoral niches and which merely represent physiological background fluctuations at the luminal level. This approach may minimize signal misinterpretation and preserve screening performance (Zhao et al., 2020; Russo et al., 2023).

The clinical generalizability of screening models is also crucial. Differences in dietary habits, comorbidities, medication exposure, and baseline microbiota across populations may affect the stability and reproducibility of fecal microbial biomarkers. Future CRC microbial diagnostic strategies should place greater emphasis on cross-cohort validation and adjustment for clinical covariates to improve model generalizability and clinical applicability. For applications requiring greater local representativeness, mucosa-adherent sampling approaches such as rectal swabs may capture mucosal-level microecological signals more accurately while retaining practical advantages in acceptability and minimal invasiveness. They may therefore offer a feasible compromise between non-invasive screening and the acquisition of local ecological information (Nejati et al., 2022; Nikolić et al., 2025).

### 4.3 Implications for the stratified design of microbiome-based interventions

From a spatial-niche perspective, microbiome-based interventions should not be understood simply as uniform modulation of the gut microbiota as a whole. Instead, intervention strategies should be stratified according to target site and pathological level into three niche-oriented intervention levels: remodeling luminal microbial community structure, regulating mucosa-colonizing microbiota, and targeted modulation of the intratumoral microecology, thereby enabling niche-specific targeting (Chen et al., 2025a; Fan et al., 2025).

Luminal-level interventions primarily aim to reshape the global intestinal microecological context and may be particularly relevant to CRC risk reduction, adjunctive treatment, and post-treatment ecological restoration. Representative strategies, including dietary modification, dietary fiber supplementation, probiotics, prebiotics, and fecal microbiota transplantation (FMT), mainly act on gut-wide microbial community structures and metabolic networks. Such interventions may indirectly influence CRC risk, treatment tolerance, and post-treatment recovery by regulating bile acid metabolism, promoting short-chain fatty acid production, and improving the overall immune-inflammatory state (Fan et al., 2025; Wang et al., 2025a; Wei et al., 2026).

Clinical evidence from other tumor types further supports the potential of luminal microbiota modulation to influence antitumor immunity. In patients with anti-PD-1-refractory metastatic melanoma, FMT followed by anti-PD-1 reinduction produced objective responses in 3 of 10 patients, including one complete response and two partial responses (Baruch et al., 2021). In another study, responder-derived FMT combined with pembrolizumab provided clinical benefit in 6 of 15 evaluable patients, including three objective responses and three cases of durable stable disease lasting more than 12 months (Davar et al., 2021). More recently, the phase 2 FMT-LUMINate trial reported objective response rates of 80% in the non-small cell lung cancer cohort receiving healthy-donor FMT plus pembrolizumab and 75% in the melanoma cohort receiving FMT plus nivolumab–ipilimumab (Duttagupta et al., 2026). Because these studies involved small, non-randomized, open-label cohorts and tumor types other than CRC, their findings should be interpreted as cross-tumor proof-of-concept rather than direct evidence of clinical efficacy in CRC. Notably, 13 of 20 patients (65%) in the melanoma cohort experienced grade 3 or higher adverse events in the setting of FMT plus dual checkpoint blockade, underscoring the importance of treatment context and prospective safety monitoring.

Mucosal-level interventions focus on correcting local ecological imbalance at the host–microbe interface. Mucosal barrier injury, aberrant adhesion of pathogenic bacteria, and biofilm colonization are critical events driving early pathological progression in CRC. Accordingly, strategies targeting mucosa-colonizing microbiota, biofilm degradation, and improvement of the local inflammatory microenvironment may offer a more direct means of modulating disease-relevant pathological processes. For example, local delivery of antimicrobial peptides, specific bacteriophages, engineered probiotics, or mucosa-targeted drug delivery systems may help regulate mucosal microecological homeostasis and interrupt microbiota-driven components of early CRC progression (Wang et al., 2023; Fan et al., 2025; Tan et al., 2025).

Interventions targeting the intratumoral microbiota represent one of the most challenging yet promising frontiers in precision therapy. Intratumoral microbiota may directly participate in drug metabolism, mediate local immunosuppression, and regulate tumor sensitivity to therapy. Therefore, their management should be incorporated into a “pharmacoecology” framework. Future directions worthy of focused exploration include targeted elimination of pathogenic bacteria within tumors, construction of engineered probiotics as living drug-delivery vehicles, development of tumor-targeted nanodelivery systems, and synergistic microecological modulation strategies combined with chemotherapy or immune checkpoint inhibitors (Han et al., 2025; Jia et al., 2025; Liao et al., 2025; Niu et al., 2025; Sanjay et al., 2025; Tan et al., 2025; Wang et al., 2025b).

### 4.4 Current research bottlenecks and emerging technological directions

Although the spatial-niche framework provides a clearer interpretive lens for CRC microbiome research, the field still faces several major bottlenecks. Most existing studies rely on bulk sequencing approaches, which can characterize overall microbial community composition but cannot preserve the spatial distribution of microorganisms within tissues. Moreover, multi-site and multi-timepoint paired studies within the same individuals remain scarce, limiting systematic reconstruction of the continuum from the intestinal lumen to the mucosa and then to the intratumoral compartment. In addition, low microbial biomass and exogenous contamination remain persistent challenges in intratumoral tissue samples, making functional validation and causal inference far more difficult than taxonomic description.

Another key practical bottleneck is the lack of standardization across studies in sampling methods, sample processing, library preparation, sequencing platforms, and bioinformatics strategies, which substantially undermines the reproducibility and cross-center generalizability of findings. This issue is particularly evident in the development of microbiome-based screening models, where cohort-specific microbial backgrounds, differences in comorbidities, and lifestyle factors often prevent models that perform well in one cohort from being directly generalized to other populations.

Emerging spatially resolved technologies provide important tools for addressing these limitations. Advanced approaches such as spatial transcriptomics, multiplex fluorescence *in situ* hybridization, mass spectrometry imaging, single-cell sequencing, and high-dimensional spatial proteomics enable microbial localization, characterization of neighboring host-cell states, and assessment of local metabolite distributions while preserving tissue architecture (Li and Luo, 2024; Chen et al., 2025b; Cheng et al., 2025; Gao et al., 2025; Shi et al., 2025a; Zhang et al., 2025; Liu et al., 2026). Therefore, future studies should not only incorporate advanced technologies such as spatial transcriptomics and *in situ* imaging, but also promote standardized sampling protocols, robust contamination-control frameworks, multicenter cross-cohort validation, and integrative multi-omics analysis. Only by making CRC microbiome research spatially resolved, functionally validated, and methodologically standardized can the field move beyond correlation-based description toward precision medicine applications that are mechanistically interpretable, experimentally verifiable, and clinically translatable.

## 5 Discussion

Overall, the spatial-niche perspective provides an important conceptual framework for understanding persistent inconsistencies and limited reproducibility in CRC microbiome research. Focusing on the three core niches—fecal, mucosal, and intratumoral—this review summarizes their microbial features, biological significance, and potential clinical value. It further proposes a “seed bank–colonizers–specialized populations” model to explain the continuous yet non-equivalent hierarchy among these niches. This framework suggests that fecal, mucosal, and intratumoral microbiota are not simple manifestations of the same microecological signal across different specimen types. Instead, they carry ecological information from distinct spatial levels during CRC initiation and progression.

This perspective helps explain seemingly contradictory findings derived from different niches in CRC microbiome research. It also indicates that the key challenge is not simply to determine which finding is correct, but to understand and integrate these differences within a spatially stratified framework. Some taxa may show strong discriminatory performance in fecal samples without necessarily being stably enriched at the tissue level. Conversely, other taxa may exhibit strong pathological relevance in tumor tissues or the intratumoral microenvironment without generating stable fecal detection signals. This does not necessarily mean that these findings are mutually contradictory. Rather, it may indicate that different specimen types capture microecological information from different layers of the disease process. Therefore, in future studies, specimen source should be treated as a core variable shaping the interpretation of findings, rather than merely as a technical choice in study design.

Although the spatial-niche framework has substantial explanatory value, current evidence has important limitations. First, whether intratumoral microbiota represent genuine and stable colonization, and how their functional boundaries should be defined, remain unclear. Exogenous contamination, interference from host-derived material, and technical noise in low-biomass samples must also be rigorously controlled. Second, the processes of microbial migration, selection, and retention across different niches remain incompletely understood, and their temporal sequence and causal links are still insufficiently defined. Most current studies still rely on cross-sectional designs, making it difficult to determine whether specific taxa drive CRC initiation and progression or represent secondary enrichment following alterations in the TME. In addition, conventional bulk sequencing approaches may underestimate the spatial heterogeneity of the microbiome. The distribution of microbial communities across different tumor regions and their relationships with local immune status, metabolic context, and therapeutic response require further clarification through spatially resolved analyses and functional validation. Therefore, while emphasizing the explanatory value of the “seed bank–colonizers–specialized populations” model, it is also important to avoid oversimplifying the relationships among the three niches as a unidirectional, linear, and universally applicable migration chain.

In summary, fecal, mucosal, and intratumoral microbiota together constitute an interconnected yet bounded spatial ecological system in CRC microbiome research. Future studies should, whenever feasible, perform multi-niche paired sampling within the same patients in prospective, multicenter, longitudinal cohorts to delineate the temporal patterns of microbial changes across niches during CRC progression. Integrating advanced approaches, including spatially resolved technologies, multi-omics analysis, strain-level tracking, and functional experiments, will further help clarify the spatial localization, ecological status, and pathological roles of key microbial taxa. Only by fully accounting for the intrinsic properties of different niches can CRC microbiome research move beyond correlation-based description toward mechanistic interpretability, reproducible evidence, and clinical translatability.
